# Image-Based Analysis of Morphometric Differences Between Sea-Caught and Farmed Large Yellow Croaker (*Larimichthys crocea*)

**DOI:** 10.3390/ani16040601

**Published:** 2026-02-14

**Authors:** Yatong Yao, Quanyou Guo, Shengmao Zhang, Junjie Wu, Tianfei Chen, Na Lin, Zuli Wu, Hanfeng Zheng

**Affiliations:** 1 Key Laboratory of Fisheries Remote Sensing, Ministry of Agriculture and Rural Affairs, East China Sea Fishery Research Institute, Chinese Academy of Fishery Sciences, Shanghai 200090, China; ytztta@163.com (Y.Y.); dhsguoqy@163.com (Q.G.); tslwjj2022@163.com (J.W.); chengtf@ecsf.ac.cn (T.C.); lina903368043@163.com (N.L.); wuzl@ecsf.ac.cn (Z.W.); 2 College of Fisheries and Life Science, Dalian Ocean University, Dalian 116023, China

**Keywords:** large yellow croaker (*Larimichthys crocea*), YOLOv11Instance segmentation, morphological differentiation, surface-area phenotypes, deep learning

## Abstract

Morphological differences between sea-caught and farmed fish reflect environmental conditions and long-term domestication. However, standardized and objective quantification of these differences remains limited for many commercially important species. The large yellow croaker (*Larimichthys crocea*) represents a typical marine fish with clear contrasts between natural and aquaculture production systems. In this study, an image-based phenotyping workflow was developed to quantify external morphological traits of sea-caught and farmed *L. crocea*. Visible-light images were acquired under standardized conditions. A YOLOv11-based instance segmentation model was applied to automatically delineate major anatomical regions, including the body, head, eyes, pectoral fins, and tail. Surface areas and proportional indices were calculated following geometric calibration to ensure measurement consistency. The segmentation model achieved high accuracy on the test dataset (mAP@50 > 98%). Morphometric analyses revealed clear differences between the two groups. Farmed individuals exhibited larger body-related surface areas, whereas the relative proportions of pectoral fins and tail regions were reduced. Sea-caught fish showed higher proportional investment in locomotor structures, consistent with the physical demands of natural marine environments. These results indicate a shift in morphological allocation associated with aquaculture, characterized by enhanced trunk growth and reduced relative development of propulsion-related structures. The proposed workflow provides a rapid, non-invasive, and reproducible approach for fish morphometric analysis. It offers practical potential for phenotypic monitoring and stock assessment, while contributing quantitative evidence for domestication-driven morphological divergence in marine fishes.

## Simple Summary

Fish raised on farms often look different from fish captured from the sea, but these differences are usually judged subjectively rather than measured objectively. In this study, we used ordinary photographs and artificial intelligence to compare the body shapes of sea-caught and farmed large yellow croaker, an important marine food fish in China. A computer program was trained to automatically outline different external body parts, such as the head, body, fins, tail, and eyes, and then measure their surface areas. The results showed that farmed fish had much larger body areas, while their fins and tails were relatively smaller. In contrast, sea-caught fish retained relatively larger fins and tails, which are important for swimming ability and survival in the open ocean. These findings indicate that farming environments with abundant food and limited space promote body mass growth, whereas natural marine conditions favor body shapes that support movement and escape. The method used in this study is fast, non-invasive, and low-cost, and does not require handling live animals. It can support fish breeding, product grading, and quality control, and also provides a practical tool for understanding how aquaculture alters fish body shape.

## 1. Introduction

The large yellow croaker (*Larimichthys crocea*) is an economically important marine fish species endemic to China. It belongs to the family Sciaenidae within the order Perciformes and is widely distributed along the coastal waters of the East China Sea, Yellow Sea, and northern South China Sea, where it is classified as a warm–temperate demersal species [1]. Owing to its favorable flesh quality, distinctive flavor, and high nutritional value, *L. crocea* has long been regarded as one of the “three famous marine fishes” in China. Since the 1950s, capture fisheries of this species once reached annual yields of several hundred thousand tons, establishing it as a key component of China’s coastal fisheries [2]. However, increasing fishing pressure, degradation of coastal habitats, and broader marine ecosystem disturbances have led to a pronounced decline in wild populations [3,4], which approached near-collapse by the early 21st century [5].

To conserve and utilize this key resource, China initiated artificial propagation and stock enhancement programs in the 1980s and achieved large-scale hatchery production in the 1990s [6]. Subsequently, large yellow croaker aquaculture expanded rapidly, forming a major coastal farming belt concentrated in Fujian and Zhejiang Provinces [7]. Recent global assessments indicate that aquaculture production surpassed capture fisheries for the first time in 2022, with China contributing more than 60% of total output [8]. Within this context, large yellow croaker has remained one of the most important marine finfish species cultured in China, playing a substantial role in supporting coastal economies and restructuring marine fisheries production systems [9].

At present, large yellow croaker harvested along the Chinese coast originate from multiple pathways, forming a heterogeneous and complex resource system. Naturally reproducing wild populations constitute the historical foundation of the fishery. Under prolonged fishing pressure and habitat degradation, however, wild stocks in major spawning grounds such as the East China Sea have been severely depleted [4], and only sporadic individuals are now encountered, primarily in marine protected areas or deeper offshore waters. Stock enhancement via large-scale release of hatchery-reared juveniles has become the dominant source of current catches, while cage escapees from near-shore mariculture systems represent an additional contribution [10]. This complexity complicates population identification and may obscure phenotypic comparisons between wild and farmed fish if not explicitly acknowledged.

In contrast, the origin of farmed large yellow croaker is relatively concentrated and derives almost exclusively from hatchery-produced juveniles. Farmed populations rely on industrial-scale artificial breeding, followed by grow-out in near-shore floating cages, set-net or pen culture systems, and, to a lesser extent, land-based recirculating aquaculture facilities [11]. These controlled production systems provide a useful reference for examining domestication-related morphological changes.

Artificial rearing environments differ substantially from natural marine habitats in ecological conditions, feed composition, and available activity space. Prolonged domestication under high-density culture has therefore promoted divergence between farmed and sea-caught populations in morphology, physiology, and behavior [12,13]. Previous studies have reported that farmed *L. crocea* typically exhibit deeper bodies and increased body height, whereas sea-caught individuals tend to be more slender, with better-developed caudal regions and darker body coloration [14,15]. Such differences reflect contrasting ecological demands and life-history strategies and may further influence product quality and swimming performance [16,17]. Despite these observations, quantitative characterization of regional morphological allocation remains limited.

Traditional studies of fish morphology have relied mainly on manual linear measurements and landmark-based geometric morphometrics [18,19]. While these methods have supported taxonomic and ecological studies, they are labor-intensive and sensitive to operator experience and measurement conditions [20]. These limitations restrict their application in large-scale analyses and routine monitoring.

Recent advances in computer vision and deep learning have enabled image-based approaches for non-contact and high-throughput morphological analysis [8,21,22,23]. Compared with manual measurements, automated image analysis improves efficiency and repeatability while reducing subjective bias. Among available frameworks, YOLO-based models have been widely applied in aquaculture and ecological monitoring owing to their balance between accuracy and computational efficiency [24,25,26,27,28,29].

Although X-ray and CT imaging have been used to investigate internal skeletal structures [30,31,32], these approaches require specialized equipment and complex workflows. In contrast, visible-light images acquired using ordinary cameras are cost-effective and suitable for large-scale deployment [33,34]. By segmenting distinct external anatomical regions, two-dimensional images allow for the extraction of area-based phenotypic traits, which provide quantitative descriptors of body shape and proportional allocation [35,36].

Area-based morphological traits have been successfully applied to growth evaluation, body-shape characterization, and product grading in fish [33,37,38]. Such traits capture population-level differences and offer practical advantages for phenotypic comparison. However, their application to systematically quantify morphological divergence between sea-caught and farmed *L. crocea* using automated deep-learning segmentation remains limited.

Morphological divergence between sea-caught and farmed large yellow croaker arises from long-term environmental adaptation and artificial selection [39]. In natural marine environments, wild individuals experience higher locomotor demands and predation pressure, favoring streamlined body shapes and well-developed fins [40,41,42]. By contrast, aquaculture environments promote increased allocation to trunk growth and body depth under reduced locomotor constraints [43,44,45]. Quantifying these differences using standardized and scalable methods is essential for germplasm identification, stock assessment, and aquaculture management.

In this study, a YOLOv11-based instance segmentation model was applied to automatically segment visible-light images of large yellow croaker and extract surface-area information for major external anatomical regions. Area proportions were used to compare two-dimensional projected morphologies between sea-caught and farmed populations. The objectives were to (i) establish an automated workflow for structure-level segmentation and area extraction; (ii) develop proportional metrics linking regional morphology to overall body shape; and (iii) evaluate morphological divergence between populations. This study aims to provide both a practical phenotyping framework and quantitative evidence for domestication-driven morphological differentiation in *L. crocea.*

## 2. Materials and Methods

### 2.1. Data Source

**Figure 1:**
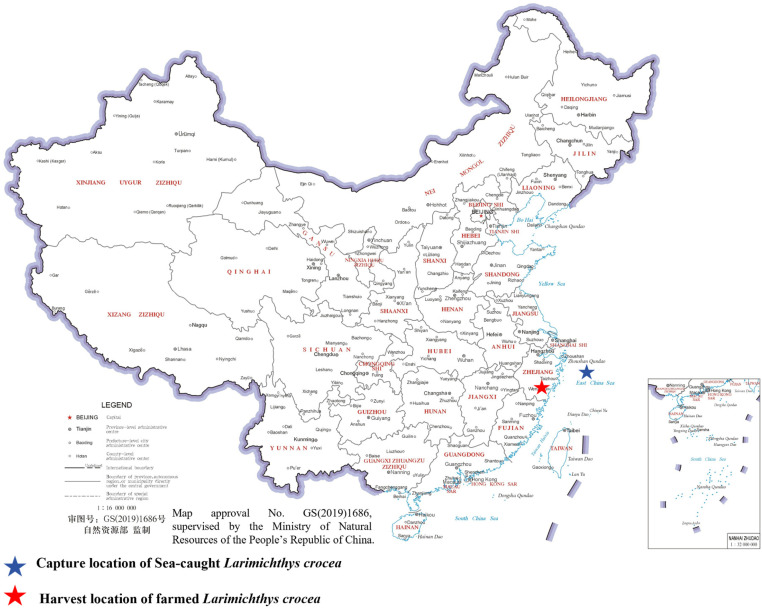
Sampling locations of sea-caught and farmed large yellow croaker (*L. crocea*). Geographic distribution of sampling locations for large yellow croaker (*L. crocea*) used in this study. The blue star indicates the capture location of sea-caught individuals from the East China Sea, while the red star represents the aquaculture region where farmed individuals were obtained. The map provides regional context for the origins of the analyzed specimens. The base map used in this study was derived from the standard map service provided by the Ministry of Natural Resources of the People’s Republic of China (http://bzdt.ch.mnr.gov.cn/, accessed on 12 January 2026), with map approval number GS(2019)1686. The base map has not been modified in any way that compromises territorial integrity, and all interpretations presented in this study are consistent with the official position of the Chinese government.

Farmed specimens used in this study were collected from commercial aquaculture sites in Fujian and Zhejiang Provinces, China, while sea-caught *L. crocea* were captured from the East China Sea (Figure 1). All individuals used in the analysis were adult fish. Following capture, all specimens were immediately transported to Wenzhou Huangyu National Group Co., Ltd. under low-temperature conditions using ice-pack refrigeration to minimize post mortem deformation and preserve body integrity prior to imaging.

A total of 270 individuals were included in the morphometric analysis, comprising 90 farmed and 180 sea-caught fish. Farmed individuals exhibited a total length of 29.41 ± 1.79 cm and a body height of 7.62 ± 0.47 cm, whereas sea-caught individuals showed a total length of 25.43 ± 3.60 cm and a body height of 6.44 ± 0.82 cm. Farmed fish were reared in intensive near-shore cage culture systems and fed formulated commercial diets throughout the grow-out period, rather than relying on natural food sources. All measurements were conducted using a standardized protocol, and measurement locations are indicated directly in the corresponding figures. Individuals within each group were selected within a relatively narrow size range to minimize the potential influence of size-dependent morphological variation on subsequent analyses.

**Figure 2:**
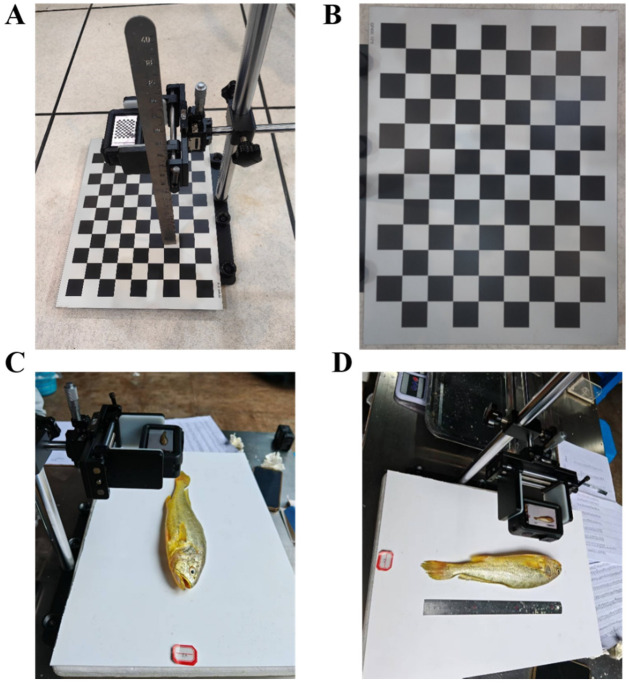
Schematic workflow of the visible-light imaging system used for *L. crocea*. (**A**) Camera intrinsic and extrinsic parameter calibration performed using a high-precision checkerboard calibration plate and a vertical reference scale, enabling correction of lens geometry and establishing the imaging coordinate system; (**B**) Frontal capture of the calibration plate for accurate lens-distortion correction and geometric rectification, ensuring consistency of pixel-to-metric mapping across all subsequent images; (**C**) Lateral imaging setup in which the camera is mounted above a standardized white background panel, illustrating the fixed shooting distance, illumination conditions, and positioning framework used to photograph individual fish specimens; (**D**) On-site imaging configuration showing the assembled camera mounting frame, adjustable sample platform, and integrated reference ruler, forming a stable and replicable imaging environment for large-scale data acquisition.

To ensure geometric accuracy and scale consistency in the visible-light images, the camera system was calibrated prior to image acquisition, as illustrated in Figure 2. Camera calibration was performed using the classical Zhang’s checkerboard method, in which a planar checkerboard was imaged from multiple viewpoints to estimate the intrinsic camera matrix and lens distortion parameters. The calibration board consisted of square cells measuring 30 mm × 30 mm. During calibration, the camera was fixed at a height of 220 mm, and a sufficient number of checkerboard images were captured from varying angles to extract stable corner features. The resulting intrinsic parameters and distortion coefficients were applied to all experimental images to correct radial and tangential distortions. This procedure ensured that the visible-light images used for instance segmentation possessed accurate spatial scales and consistent geometric properties, thereby supporting reliable extraction of area-based morphometric traits.

### 2.2. Data Acquisition

**Figure 3:**
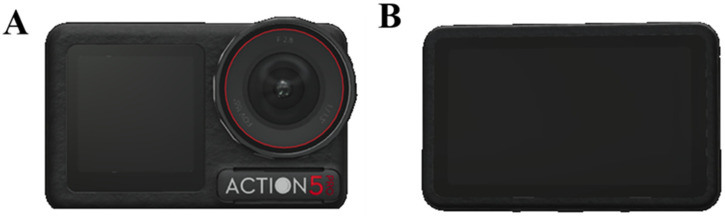
Visible-light imaging device. (**A**) Front view of the DJI Action 5 Pro camera used for image acquisition, illustrating the optical lens module and compact waterproof housing; (**B**) Rear interface showing the real-time preview screen and adjustable shooting parameters. The device is equipped with a 1/1.3-inch CMOS sensor, supports 4K/120 fps high-frame-rate recording with high dynamic range, and provides enhanced low-light imaging performance and robust environmental resistance, enabling stable data collection under field conditions.

A DJI Action 5 Pro camera (DJI, Shenzhen, China, Figure 3) was used for visible-light image acquisition in this study. The camera is equipped with a 1/1.3-inch CMOS sensor and a wide-angle lens, enabling high-resolution imaging under variable lighting conditions. After geometric calibration, the effective spatial resolution was approximately 140 μm, which was sufficient for accurate documentation of external morphological features.

To improve dataset diversity and model robustness, each *L. crocea* specimen was photographed under multiple orientations and illumination conditions. In total, 720 visible-light images were acquired, including 360 images of sea-caught individuals and 360 images of farmed individuals. All images were collected under standardized exposure settings (ISO 100–200; shutter speed 1/250–1/500 s) and underwent color correction prior to subsequent instance segmentation and morphometric analysis.

### 2.3. Dataset Construction

**Figure 4:**
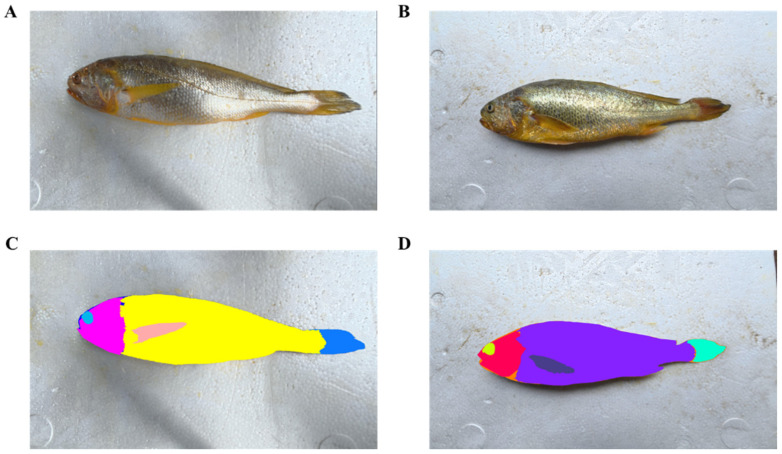
Image dataset construction and manual annotation process. (**A**,**C**) Representative raw visible-light images of sea-caught and farmed *L. crocea* acquired from different orientations. (**B**,**D**) Manual pixel-level segmentation and annotation of key anatomical regions using Roboflow and CVAT. A total of twelve target classes were defined, covering both farmed and sea-caught groups: body, eyes, head, pectoral fin, tail, and whole fish. Each class was assigned a fixed color label for visualization and consistency across the dataset. The color–class mappings were as follows: Farmed fish: Body (purple), Eyes (lime), Head (red), Pectoral fin (navy), Tail (cyan), Whole fish (orange); Sea-caught fish: Body (yellow), Eyes (light blue), Head (magenta), Pectoral fin (pink), Tail (royal blue), Whole fish (dark blue). All segmentation masks were annotated and cross-checked by the same trained operator to ensure intra-observer consistency and dataset reliability.

A total of approximately 1088 fish images were acquired using the calibrated camera system (Figure 4A,C). After removing invalid or low-quality images, instance segmentation yielded 6528 individual objects. From these objects, visible-light images depicting *L. crocea* from different orientations were screened, and the subset required for model prediction was selected for manual annotation.

Because the analysis focused on twelve anatomical categories—Cultivation Head, Cultivation Eyes, Cultivation Tail, Cultivation Pectoral Fin, Cultivation Body, Cultivation Total, Wild Head, Wild Eyes, Wild Tail, Wild Pectoral Fin, Wild Body, and Wild Total—each region of interest was manually annotated. Annotation was performed using the open-source tools Roboflow (https://roboflow.com, assessed on 20 December 2025) and CVAT (Computer Vision Annotation Tool), and representative annotation results are shown in Figure 4B,D.

To ensure consistency and minimize human-induced variability, all annotations were performed by a single annotator and subsequently reviewed and validated by another trained reviewer. After annotation, the dataset was randomly partitioned into training, validation, and test sets in a ratio of 7:2:1 for subsequent model training and performance evaluation.

### 2.4. Instance Segmentation and Area Calculation

After model training, the optimized weight parameters were applied to perform instance segmentation on the target images. Based on the segmentation outputs, the areas of different anatomical regions of *L. crocea* were calculated, followed by an assessment of measurement errors and their potential sources. Using these instance-level segmentation results, area metrics were extracted for the following twelve categories: Cultivation Head, Cultivation Eyes, Cultivation Tail, Cultivation Pectoral Fin, Cultivation Body, Cultivation Total, Wild Head, Wild Eyes, Wild Tail, Wild Pectoral Fin, Wild Body, and Wild Total.

**Figure 5:**
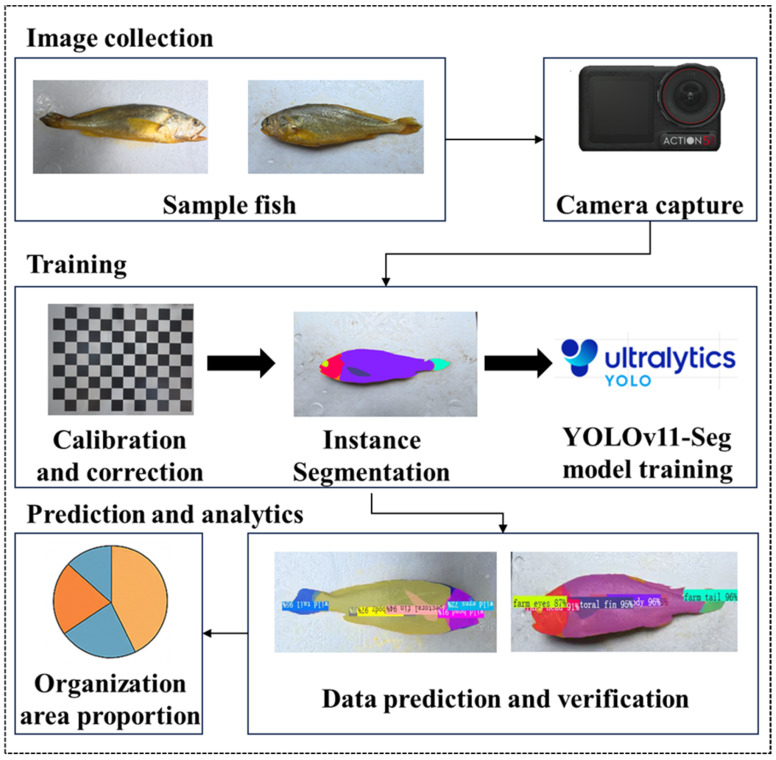
Workflow of area-based phenotypic measurement using YOLOv11-Seg. Schematic illustration of the automated “mask–index–threshold” morphometric pipeline. The workflow consists of five major stages: (1) Image acquisition, where high-resolution visible-light images of *L. crocea* are collected using the DJI Action 5 Pro camera under standardized lighting and background conditions; (2) Instance segmentation annotation, during which fish body regions are manually labeled in Roboflow and exported as segmentation masks; (3) Model training, where the YOLOv11-Seg network is trained using annotated masks to learn multi-region morphological features; (4) Prediction and mask generation, enabling automatic identification and segmentation of key anatomical structures in new samples; (5) Area-based phenotypic analysis, in which segmentation masks are converted into pixel-area measurements, followed by the computation of body-region area ratios and statistical comparisons between sea-caught and farmed populations. This workflow provides a fully automated and replicable path from raw image acquisition to quantitative morphometric inference.

Subsequently, a machine learning–based predictive model was constructed to estimate the proportional area contributions of each anatomical region. Visible-light images were used as the input data, and the predicted area ratios were generated accordingly. The complete workflow of this process is illustrated in Figure 5.

Because visible-light images capture only the lateral projection of the fish body and cannot provide information on body thickness, this study focused exclusively on the lateral surface areas of the twelve anatomical categories listed above. These lateral-area measurements served as the input features for the predictive modeling of area proportions.

### 2.5. Instance Segmentation Model

Object detection and instance segmentation are increasingly used in fisheries and aquaculture research for automated phenotypic analysis. Among existing approaches, the YOLO family has been widely adopted because of its efficient end-to-end inference and suitability for real-time applications. The latest version, YOLOv11, further improves detection and segmentation performance while maintaining computational efficiency. Its segmentation variant, YOLOv11-Seg, enables pixel-level delineation of target regions and is well-suited for extracting external morphological features from fish images.

Recent studies have demonstrated the applicability of instance segmentation in aquaculture systems. For example, Mask R-CNN has been applied to fish-body segmentation and length estimation in marine cage culture [46], while the Aquabyte system integrates instance segmentation to support automated sea lice detection in Atlantic salmon (*Salmo salar*) farming [47,48]. Lightweight segmentation models have also been used for real-time monitoring of fish behavior and population dynamics in high-density culture environments. These applications highlight the growing role of instance segmentation as a practical tool for fish health monitoring, production management, and resource assessment.

**Figure 6:**
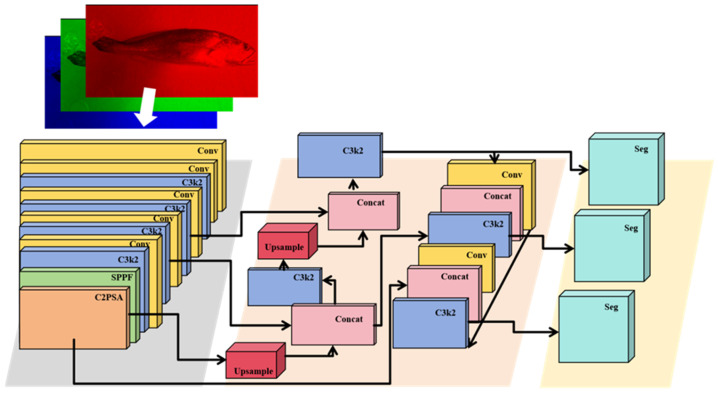
YOLOv11-Seg network architecture. The model comprises four major components: (1) Input, which receives standardized images after preprocessing and normalization; (2) Backbone, implemented using an enhanced CSPDarknet framework to extract multi-scale spatial–semantic features with improved gradient flow and computational efficiency; (3) Neck, integrating Feature Pyramid Network (FPN) and Path Aggregation Network (PAN) modules to fuse deep and shallow feature maps, thereby strengthening cross-scale representation for small and large target regions; and (4) Segmentation head, which performs bounding-box regression, object classification, and pixel-level instance mask prediction through a dedicated mask branch. This architecture enables high-resolution feature extraction, robust multi-region discrimination, and precise delineation of anatomical boundaries, supporting reliable morphological segmentation of *L. crocea* under varied imaging conditions.

Based on this background, the present study employs the YOLOv11-Seg framework for automated instance segmentation of fish bodies. A schematic overview of the model is provided in Figure 6. Detailed descriptions of network architecture and internal modules are provided only to the extent necessary for methodological transparency and reproducibility.

Model training was performed on a Windows 11 platform using Python 3.10.16 and PyTorch 2.2.2, with acceleration provided by an NVIDIA RTX 4070 GPU(NVIDIA, Santa Clara, CL, USA) and CUDA version 12.5. The batch size was set to 8, and the initial learning rate was 0.001, optimized using the Adamax optimizer. The input image resolution was 8000 × 4512 pixels. Based on YOLO predictions, pixel-level segmentation outputs were converted into physical area measurements, enabling extraction of 12 anatomical segmentation categories for *L. crocea*. Model performance was evaluated using standard metrics, including Precision (P), Recall (R), mean Average Precision at an IoU threshold of 0.5 (mAP@50), and mean Average Precision across IoU thresholds from 0.5 to 0.95 (mAP@50–95), with the corresponding definitions provided below.

(1) $$Precision=\frac{TP}{\left(TP+FP\right)}$$

(2) $$Recall=\frac{TP}{\left(TP+FN\right)}$$

(3) $$AP=\int_{0}^{1}Precision\left(r\right),dr$$

(4) $$mAP_{\left\{@0.5\right\}}=\frac{1}{N_{\left\{classes\right\}}}\sum_{{\left\{i=1\right\}}_{\left\{i@0.5\right\}}^{\left\{N_{\left\{classes\right\}}\right\}AP}}$$

(5) $$mAP_{\left\{@0.5-0.95\right\}}=\frac{1}{N_{\left\{classes\right\}}}\sum_{{\left\{i=1\right\}}_{\left\{i@0.5-0.95\right\}}^{\left\{N_{\left\{classes\right\}}\right\}AP}}$$

In Equations (1) and (2), TP, FP, and FN represent the numbers of true positives, false positives, and false negatives, respectively. Precision (P) describes the proportion of correctly predicted positive samples among all predicted positives and reflects the reliability of positive predictions. Recall (R), also referred to as sensitivity, represents the proportion of actual positive samples that are correctly identified and characterizes the model’s ability to detect target instances.

The metrics mAP@50 and mAP@50–95 denote the mean Average Precision calculated at Intersection-over-Union (IoU) thresholds of 0.5 and from 0.5 to 0.95, respectively. These indicators summarize overall detection performance across categories under different localization strictness levels. In particular, mAP@50–95 provides a more stringent and comprehensive evaluation of the model’s localization accuracy under high-precision requirements.

### 2.6. Instance Area Calculation Method

To achieve accurate area quantification of the anatomical structures of *L. crocea*, Zhang’s checkerboard calibration method was employed to geometrically calibrate the visible-light imaging system. Based on the estimated intrinsic camera parameters, a conversion relationship was established between pixel dimensions and real-world measurements. The checkerboard used for calibration consisted of square cells with physical dimensions of 30 mm × 30 mm, and the camera was positioned at a fixed height of 220 mm during the procedure.

1. Estimation of Camera Intrinsic Parameters

During calibration, checkerboard images were captured from multiple viewpoints, and corner features were extracted to construct planar homography matrices. These homography relations were then used to solve for the intrinsic camera matrix M, whose general form can be expressed as

$$M=\left[\begin{array}{ccc} f_{x} & 0 & u_{0}\\ 0 & f_{y} & v_{0}\\ 0 & 0 & 1 \end{array}\right]=\left[\begin{array}{ccc} \frac{f}{d_{x}} & 0 & u_{0}\\ 0 & \frac{f}{d_{y}} & v_{0}\\ 0 & 0 & 1 \end{array}\right]$$

where $f_{x},f_{y}$ represent the effective focal lengths in the pixel coordinate system; $u_{0},v_{0}$ denote the principal point coordinates; and $d_{x},d_{y}$ correspond to the physical pixel sizes (mm/pixel). This intrinsic matrix establishes the geometric mapping between pixel coordinates and real-world physical space.

2. Scale Factor Estimation Using the Checkerboard Pattern

With the known physical edge length of each checkerboard square L= 30 mm= 3.0 cm, the corresponding pixel length *l*_*p*_ of the same edge can be measured in the distortion-corrected image. According to the imaging geometry, the scale conversion factor can be expressed as

(6) $$s=\frac{L}{l_{p}}\left(\mathrm{cm}/\mathrm{pixel}\right)$$

Furthermore, if the pixel area of an instance-segmented region is denoted by $a_{\mathrm{region}}$, its corresponding real-world area can be expressed as

(7) $$A_{\mathrm{region}}=s^{2}\cdot a_{\mathrm{region}}\;$$

This calibration approach uses the known physical dimensions of the checkerboard as geometric constraints and, together with distortion-corrected images, ensures stable scale accuracy even when shooting distance and camera pose vary. Compared with single-frame scale inference methods (e.g., coin-based calibration), the checkerboard-based calibration offers several advantages: stronger geometric constraints that simultaneously allow for estimation of intrinsic parameters, extrinsic parameters, and distortion coefficients across multiple viewpoints; improved stability of pixel scale after distortion correction; and consistent area measurement accuracy under different imaging conditions. All subsequent area calculations in this study—including those for the body, head, eyes, pectoral fin, and tail regions—were converted from pixel area to real-world area using Equations (6) and (7).

### 2.7. Data Processing

Based on the calibrated scale factors, the trained YOLO instance segmentation model was applied to the experimental images to perform inference and generate binary masks $\left\{M_{i}\right\}$ corresponding to the target categories (Cultivation Head, Cultivation Eyes, Cultivation Tail, Cultivation Pectoral Fin, Cultivation Body, Cultivation Total, Wild Head, Wild Eyes, Wild Tail, Wild Pectoral Fin, Wild Body, and Wild Total). For any given category l, the number of pixels belonging to that region can be computed as

(8) $$N_{l}=\sum_{i=1}^{N}\;I\left(c_{i}=l\right)\;\sum_{x,y}M_{i}\left(x,y\right)\;\;$$

where $I\left(\cdot \right)$ denotes the indicator function and $c_{i}$ Incorporating the scale factor s, the real-world area of each category can be computed as

(9) $$A_{l}=N_{l}\cdot s^{2}\;\left({\mathrm{cm}}^{2}\right).$$

Furthermore, to compare the relative sizes of different anatomical structures, the area ratio for each category was calculated as follows:

(10) $$p_{l}=\frac{A_{l}}{\sum_{l^{\prime }}A_{l^{\prime }}}\times 100\backslash \%.$$

All area-based morphological variables and proportional indices were summarized as mean ± standard deviation (SD). Prior to statistical comparison, data were examined for normality using the Shapiro–Wilk test and for homogeneity of variances using Levene’s test. When assumptions of normality and homoscedasticity were satisfied, differences between sea-caught and farmed groups were evaluated using one-way analysis of variance (ANOVA). For variables that did not meet parametric assumptions, non-parametric alternatives were applied. Statistical significance was assessed at a threshold of *p* < 0.05. All statistical analyses were performed using standard statistical software, and all tests were two-tailed.

### 2.8. Error Control

To ensure that the checkerboard-based scale conversion remained accurate and reproducible under varying experimental conditions, several quality control measures were implemented during image processing and calibration:

1. Corner detection reliability: Images in which checkerboard corners could not be stably or completely detected—due to uneven illumination, occlusion, or excessive viewing angles—were excluded from calibration to prevent bias in intrinsic parameter estimation and scale factor computation.
2. Repeated measurements: During calibration, the pixel lengths or pixel areas of multiple checkerboard cells were repeatedly measured, and their mean value was used as the final scale parameter to minimize single-frame measurement error.
3. Distortion correction verification: If distortion correction produced excessive stretching or if corner reprojection errors exceeded the predefined threshold, the checkerboard images were reacquired and recalibrated to ensure the stability of the intrinsic camera matrix MMM.
4. Instance segmentation validity: During segmentation, images for which the model failed to return mask information or produced incomplete masks were excluded to avoid area estimation bias caused by missing regions.
5. Label consistency: Area conversion strictly adhered to the predefined class-label dictionary to prevent mismatches between predicted categories and their corresponding anatomical structures.

Through these error-control procedures, the checkerboard calibration, distortion correction, instance segmentation, and area conversion processes maintained high stability, scientific rigor, and reproducibility throughout the entire experimental workflow.

## 3. Results

### 3.1. Instance Segmentation Performance

To evaluate the effectiveness of the proposed YOLOv11 model, its performance was compared with several mainstream object detection and instance segmentation algorithms (Table 1). Model performance was assessed using Precision, Recall, mAP@50, and mAP@50–95, with mAP@50–95 reflecting localization accuracy across multiple IoU thresholds.

**Figure 7:**
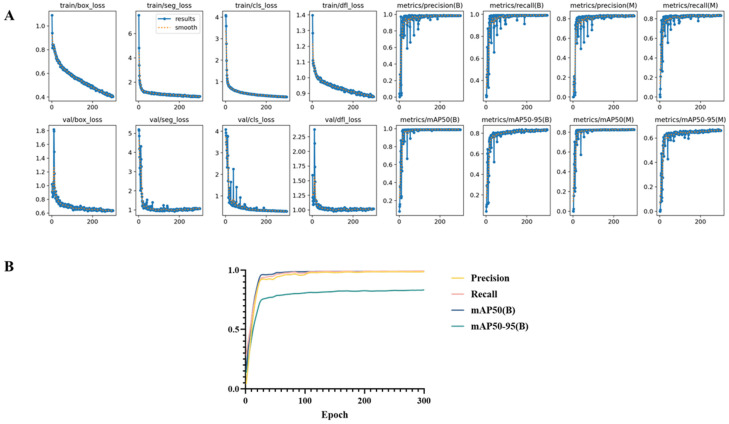
Training performance and convergence behavior of the YOLOv11-Seg model. (**A**) Training results of the YOLOv11-Seg model, illustrating the evolution of loss functions and evaluation metrics across training epochs. The curves show the optimization process for bounding box regression loss, segmentation loss, classification loss, and distribution focal loss, together with corresponding precision, recall, and mean average precision (mAP) metrics. (**B**) Training and validation curves of the YOLOv11-Seg model during optimization. The loss curves demonstrate a rapid decrease and subsequent stabilization for both training and validation datasets, indicating effective convergence and stable learning behavior. Precision and recall for both bounding-box detection and instance segmentation increase consistently during training and reach high and stable levels in later epochs, reflecting reliable detection accuracy and completeness. Overall, the smooth convergence of loss and evaluation metrics suggests good model generalization without evident overfitting.

As shown in Table 1, YOLOv11 achieved the highest overall performance, reaching a Precision of 98.53%, a Recall of 99.24%, an mAP@50 of 98.97%, and an mAP@50–95 of 83.24% (Figure 7). Compared with YOLOv5, DETR, and YOLOv8, YOLOv11 showed improved localization accuracy, particularly at higher IoU thresholds. The model also provided reliable instance segmentation results for *L. crocea*, enabling accurate contour delineation and area estimation. Representative segmentation examples are presented in Figure 7, demonstrating that the model offers sufficient accuracy and stability to support subsequent morphometric analyses.

### 3.2. Segmentation Data Analysis

**Figure 8:**
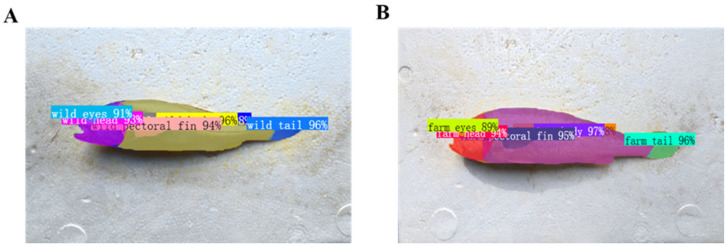
Instance segmentation results of sea-caught (**A**) and farmed (**B**) *L. crocea*. Representative outputs generated by the YOLOv11-Seg model, illustrating accurate instance segmentation of key anatomical regions—including body, head, pectoral fin, tail, and eyes—across samples with varying orientations and lighting conditions. All predicted masks follow the predefined color–class mapping established during dataset construction to ensure consistency in visual interpretation: Farmed fish: Body (purple), Eyes (lime), Head (red), Pectoral fin (navy), Tail (cyan), Whole fish (orange);Sea-caught fish: Body (yellow), Eyes (light blue), Head (magenta), Pectoral fin (pink), Tail (royal blue), Whole fish (dark blue). The results demonstrate reliable differentiation of anatomical structures between farmed and sea-caught individuals, supporting downstream morphometric quantification and area-ratio analysis.

All segmentation outputs and corresponding measurements generated in this study (Figure 8) were exported in two complementary formats to ensure data traceability, structured management, and reusability across subsequent analytical workflows. First, key parameters from the image-processing pipeline were stored in JSON files, including the image scale factor, pixel-to-physical conversion coefficients derived from checkerboard calibration, the total pixel count of each anatomical category, and the corresponding real-world area values. The hierarchical and extensible nature of JSON enables seamless integration with downstream processing scripts, iterative model training, and large-scale automated analysis.

In parallel, CSV files were generated to record essential fields such as image filename, scale factor, anatomical category, pixel counts, and calculated area values. This tabular format facilitates direct import into statistical platforms such as SPSS, R, and Python, enabling variance analysis, regression modeling, and quantitative assessment of morphological differentiation. The dual-format export strategy not only strengthens data standardization and management but also provides consistent, machine-readable inputs for large-sample statistical analyses, model error decomposition, feature-importance evaluation, and subsequent machine learning applications.

To further validate the stability and reproducibility of the entire analytical pipeline, a blind-sample evaluation was conducted. A total of 122 randomly selected *L. crocea* individuals were processed to assess the accuracy of checkerboard-based area conversion, the robustness of the instance segmentation model, and the end-to-end performance of the proposed “mask–metric–threshold” workflow.

### 3.3. Surface-Area Allocation in Wild Large Yellow Croaker

**Figure 9:**
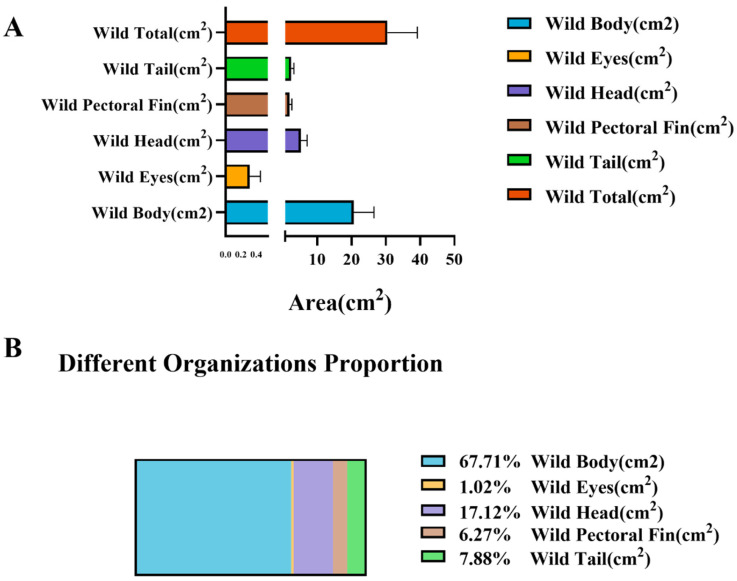
Proportional composition of external body regions in sea-caught *L. crocea*. The (**A**) shows the relative surface-area contributions of five anatomical regions (body, head, eyes, pectoral fin, and tail). The (**B**) bar chart presents mean area ratios, and the pie chart illustrates their proportional distribution.

As shown in Figure 9 and Table 2 the proportional surface-area distribution of different anatomical regions in wild large yellow croaker exhibits a pronounced hierarchical pattern that reflects functional differentiation. The body region constitutes the largest proportion, accounting for approximately 65% of the total surface area. This dominant allocation underscores its central role in maintaining overall body shape, storing energy reserves, and generating propulsive forces during locomotion.

The head region represents roughly 20% of the total area and is primarily associated with feeding, sensory acquisition, and neural control. Its relatively stable proportion suggests strong structural conservation driven by ecological niche requirements. The tail and pectoral fins collectively account for about 15% of the surface area and correspond to structures responsible for propulsion, maneuverability, and posture regulation. The preservation of these proportions indicates that wild fish possess strong swimming performance and directional control capabilities, enabling them to navigate the dynamic hydrodynamic environments of offshore waters. The eye region contributes less than 2% of the total surface area, consistent with its specialized role in visual perception rather than load-bearing or locomotor function.

Overall, the area-allocation pattern of wild large yellow croaker conforms to the typical morphology of high-mobility marine fishes: a well-developed trunk, relatively elongated caudal region, enlarged pectoral fins, and a moderately proportioned head. This structural configuration reflects the combined selective pressures of predation risk and energetic constraints in natural habitats, which drive individuals toward an optimized balance between locomotor efficiency and metabolic expenditure.

The quantified body-area proportions of wild individuals not only illuminate the functional coordination between morphology and ecological performance but also provide a reference framework for subsequent comparisons with farmed populations. Future analyses contrasting wild and cultured stocks will help elucidate the respective contributions of environmental pressures and artificial selection to morphological divergence, offering deeper insights into the adaptive morphology and ecological behavior of this species.

### 3.4. Surface-Area Allocation in Farmed Large Yellow Croaker

**Figure 10:**
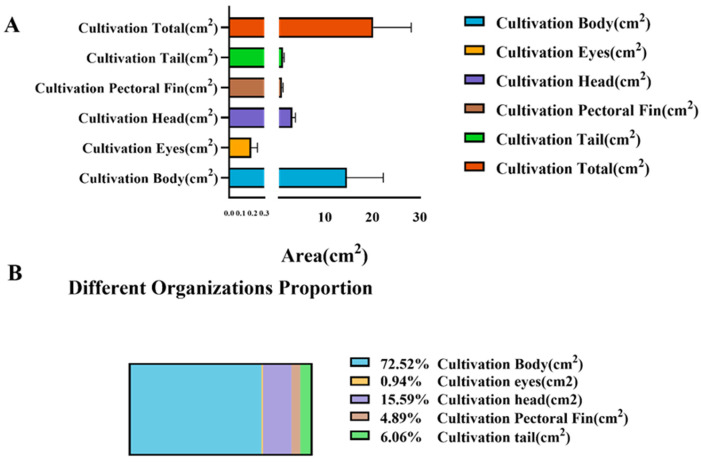
Proportional composition of external body regions in farmed *L. crocea*. The (**A**) shows the relative surface-area contributions of five anatomical regions (body, head, eyes, pectoral fin, and tail) in farmed individuals. The (**B**) bar chart presents mean area ratios for each region, and the pie chart illustrates their proportional distribution.

As shown in Figure 10 and Table 2, the surface-area allocation among different anatomical regions of farmed *L. crocea* exhibits a distinct pattern characterized by morphological centralization. The body region accounts for approximately 70% of the total surface area—a substantial increase compared with wild individuals—indicating enhanced functional emphasis on somatic growth and energy storage. The head region comprises roughly 16–18% of the surface area, slightly lower than that of wild fish, suggesting a degree of structural simplification in feeding and sensory organs under cultured conditions. The combined area proportion of the tail and pectoral fins is approximately 10–12%, representing a noticeable reduction relative to wild fish and reflecting diminished selective pressure for locomotor performance and escape ability in low-flow, high-density aquaculture environments. The eye region constitutes less than 2% of the total surface area, consistent with its specialized visual function.

Overall, the surface-area distribution in farmed *L. crocea* reflects a typical “high energy storage–low mobility demand” morphological strategy. The expansion of the body proportion illustrates a growth trajectory shaped by artificial selection and abundant nutritional input, favoring rapid somatic growth and increased body mass. In contrast, the reduced proportional areas of the tail and pectoral fins—structures closely associated with swimming efficiency and maneuverability—indicate a relaxation of ecological pressures related to sustained swimming and predator avoidance. This pattern stands in sharp contrast to the high-mobility, hydrodynamically optimized morphology observed in wild fish, highlighting a clear divergence driven by environmental differences and domestication-related selective forces.

In summary, the pronounced enlargement of the body-area proportion in farmed fish not only reveals the reshaping of morphological growth strategies under artificial conditions but also reflects the phenotypic plasticity resulting from the combined influences of environmental factors, husbandry practices, and genetic selection. These findings provide quantitative evidence for morphological differentiation between wild and farmed populations and offer important insights into body-shape divergence and energy-allocation mechanisms during the domestication process in marine fishes.

### 3.5. Morphological Differences Between Wild and Farmed Populations

**Figure 11:**
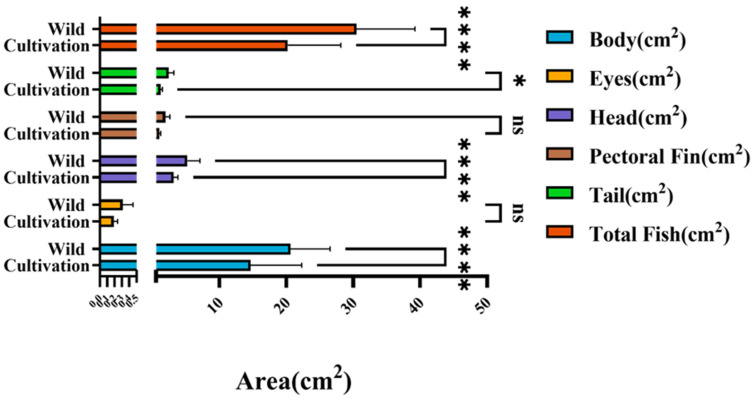
Comparison of absolute surface areas of external anatomical regions between sea-caught and farmed *L. crocea*. The figure compares the absolute surface areas of six external anatomical regions—body, head, eyes, pectoral fins, tail, and total projected area—between sea-caught and farmed populations. Farmed individuals exhibited significantly larger body area and total surface area than sea-caught fish (*p* < 0.0001), with increases of approximately 30–40%. Head area was also significantly enlarged in farmed fish (*p* < 0.0001). In contrast, the pectoral fin and eye areas did not differ significantly between groups, while the tail area showed a modest but significant reduction in farmed individuals (*p* = 0.0400). Error bars represent mean ± SD. Asterisks indicate levels of statistical significance (* *p* < 0.05; **** *p* < 0.0001; ns, not significant).

Based on the quantitative morphometric measurements derived from instance segmentation, farmed and wild populations of *L. crocea* exhibited systematic differences in both absolute area and relative anatomical composition (Figure 11 and Table 3). Six key anatomical regions—the body, head, eyes, pectoral fins, tail, and whole fish—were evaluated to assess how artificial domestication and culture environments shape morphological allocation.

The results demonstrate markedly higher absolute values for body area and total surface area in farmed individuals, with substantial differences in bar heights clearly distinguishing the two groups. This indicates that farmed fish possess larger overall body size and more pronounced muscular development of the trunk, consistent with the high-nutrient supply and rapid-growth characteristics of aquaculture systems.

Among all regions, the body area exhibited the most significant increase in farmed fish, with mean values approximately 30–40% higher than those of wild individuals—reflecting a clear trend toward trunk hypertrophy. This enlargement is likely linked to long-term artificial selection for weight gain and the stability of feeding conditions in cultured environments. Head area differed only slightly between the two groups, showing a modest increase without a consistent trend, whereas eye area remained relatively stable and accounted for less than 2% of the total area, reflecting its specialized role in light perception and its minimal sensitivity to growth-related changes. These patterns suggest that the sensory system is less influenced by aquaculture-driven selective pressures.

In contrast, the areas of the pectoral fins and tail were smaller in farmed individuals and increased at a much lower rate than the body region. These structures contribute disproportionately more to the surface-area composition of wild fish, highlighting their adaptive importance for propulsion and maneuverability in natural marine environments. Reduced proportions in cultured individuals reflect the diminished selection pressure for sustained swimming and escape performance in low-flow, high-density rearing conditions, representing a typical shift toward a “low mobility–high energy storage” morphology. The substantial increase in whole-fish area further supports the overall trend of body-size expansion in cultured stocks.

Collectively, these results confirm that farmed *L. crocea* are characterized by enlarged body regions with higher muscle allocation and simplified locomotor structures, whereas wild fish display more streamlined morphologies with greater proportional development of the pectoral and caudal fins—traits associated with enhanced swimming efficiency and escape ability. This contrast reflects the dual influences of ecological adaptation and artificial selection: mobility is the primary survival strategy in natural environments, whereas energy accumulation and growth efficiency become dominant selective forces under aquaculture conditions.

The “body amplification–locomotor simplification” pattern observed in farmed individuals illustrates a profound reshaping of energy allocation and growth strategies caused by domestication. Meanwhile, the morphological configuration of wild fish conforms to that of high-mobility species with strong hydrodynamic performance. These findings reveal a clear trade-off between growth and mobility and demonstrate how resource allocation across functional structures shifts directionally during long-term domestication. Overall, the significant enlargement of body size and reduction in locomotor structures in farmed *L. crocea* provide quantitative evidence of phenotype divergence between wild and cultured populations and offer methodological and conceptual insights into the mechanisms underlying domestication-driven morphological evolution.

### 3.6. Blind-Sample Identification Accuracy

**Figure 12:**
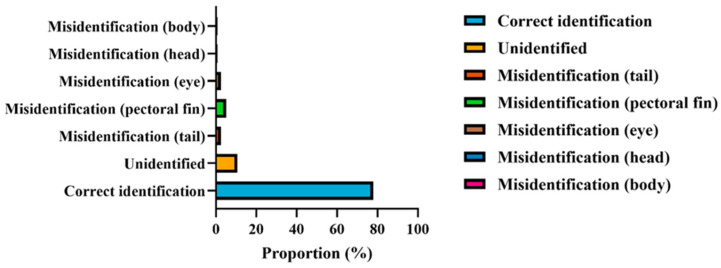
Distribution of prediction outcomes for the YOLOv11 model on blind validation samples. Bar chart illustrating the proportion of different recognition outcomes, including correct identification, unidentified samples, and misclassification of specific anatomical regions (tail, pectoral fin, eye, head, and body). The model achieved a correct identification rate of ~78%, with most errors concentrated in unidentified samples and small-structure misidentification, particularly the tail and pectoral fin. These results highlight the model’s strong performance on major body regions and its relative sensitivity to fine-scale anatomical boundaries.

In the independent validation performed on the blind-sample dataset, the YOLOv11 model demonstrated generally robust recognition performance, although a distinct pattern of structural error distribution was observed (Table 4, Figure 12). Among the 122 blind samples, completely correct identification was achieved for 95 individuals, corresponding to an overall accuracy of 77.87%. As shown in Figure 12, most errors were associated with unidentified samples and misclassification of small anatomical structures, particularly the tail and pectoral fin, whereas major body regions were identified with higher accuracy.

It should be noted that the segmentation performance (mAP) reflects pixel-level delineation accuracy of anatomical regions under supervised conditions, whereas blind-sample identification represents a higher-level classification task that integrates multiple area-based traits and is inherently influenced by biological variability among individuals. Consequently, high segmentation accuracy does not necessarily translate into equally high identification accuracy. Overall, these results indicate satisfactory generalization on previously unseen data, while also highlighting the need for further improvement to meet more stringent application requirements.

Regarding error types, unrecognized samples accounted for 11.47% (14/122), representing one of the primary contributors to overall error. This suggests that for certain individuals or specific postures, model confidence may be insufficient or the anatomical features may not be consistently expressed. For region-specific errors, misidentification of the tail and fins occurred in 2.46% (3/122) and 4.92% (6/122) of samples, respectively, totaling 7.38%. These structures are characteristically slender, morphologically complex, and functionally associated with propulsion and maneuverability, their boundaries are prone to degradation due to posture changes, partial occlusion, and weak grayscale gradients, making them more susceptible to boundary displacement and label confusion.

Misclassification of small, high-contrast structures such as the eyes occurred in 2.46% (3/122) of cases, whereas head-region errors were minimal at 0.82% (1/122). This reflects the model’s strong robustness when processing regions with stable geometric shape and clearer structural definition, such as the head contour. Notably, the body region exhibited only 0.82% (1/122) misidentification, demonstrating that detection and segmentation of large-scale primary structures remain highly reliable. Overall, the majority of errors were localized to fine or edge-complex accessory structures rather than the main body mass.

Taken together, these findings indicate that the model performs accurately for most individuals in blind-sample evaluation and exhibits high reliability in delineating global contours and major anatomical regions. However, structures associated with propulsion and maneuverability (tail and fins), as well as small-scale targets (eyes), remain the primary sources of misclassification. Future improvements may include targeted augmentation of these challenging anatomical categories, incorporation of enhanced multi-scale feature extraction and boundary-refinement modules, and optimization of confidence thresholds and post-processing strategies. Such refinements are expected to reduce the rate of non-recognition and region-specific misclassification, thereby improving the model’s engineering applicability and the precision of morphometric analyses.

## 4. Discussion

### 4.1. Applications of Instance Segmentation for L. crocea

Instance-segmentation-based image phenotyping provides a reusable and scalable framework for structured morphological quantification in *L. crocea*. Its core strength lies in supporting phenotypic measurements across multiple levels, including individuals, anatomical regions, and populations. Once segmentation masks are generated, surface area, proportional indices, and shape-related parameters can be directly derived for the whole body and for specific regions such as the body, head, pectoral fins, tail, and eyes. These measurements can be integrated into standardized phenotypic databases, enabling cross-batch, cross-population comparisons and retrospective analyses. Public datasets and benchmarks, such as DeepFish, have demonstrated the effectiveness of mask-level annotations for fish phenotyping tasks [21,49].

The same workflow is applicable to commercial scenarios requiring size estimation and grading. In fish markets and processing lines that use trays or conveyor belts, instance segmentation can be incorporated into integrated pipelines for segmentation, length measurement, weight estimation, and grading, allowing high-throughput, online statistical analysis and automated size classification. Existing industrial systems have already achieved centimeter-level or higher accuracy in fish measurement and classification tasks [50].

This approach also facilitates population discrimination and origin tracing. By combining region-composition indices (e.g., Body/Total and Tail/Total ratios) with multivariate discriminant analyses, wild, farmed, and stock-enhanced recaptured individuals can be differentiated, providing quantitative support for resource assessment, escapee identification, and regulatory management. The recent availability of mask-level datasets for fish imaged on trays or under low-visibility conditions has further improved the robustness and accuracy of instance-segmentation-based identification.

Instance-segmentation-based phenotyping supports non-invasive monitoring of health and welfare in aquaculture systems. Computer vision techniques have been applied to assess activity patterns, schooling behavior, and abnormal states. When instance segmentation is combined with detection and tracking, time-series evaluations of body condition can be generated, enabling early detection of growth deviations or acute stress responses. This capability aligns with the broader development of precision aquaculture and intelligent fisheries management [51].

Regarding model selection, both YOLO-based architectures and two-stage frameworks such as Mask R-CNN are suitable for fish segmentation tasks. Recent advances indicate that lightweight YOLO-based segmentation models offer an effective balance between accuracy and real-time performance, making them well-suited for production deployment. Emerging workflows that integrate self-iterative annotation strategies with instance segmentation further reduce annotation costs and facilitate dataset expansion for fish-specific applications.

The application of instance-segmentation-based phenotyping to *L. crocea* is both feasible and impactful. It provides quantitative tools for morphological analysis, population differentiation, and assessment of environmental effects in scientific research, while enabling real-time quality control, size grading, and origin identification in industrial contexts. Together, these advances support the development of a closed-loop “mask–metric–threshold–decision” framework that links academic research with production-level management.

### 4.2. Strengths and Limitations of the Research Method

In this study, a YOLOv11-based instance segmentation framework was applied to automatically extract masks and quantify surface areas of major external anatomical regions in wild and farmed *L. crocea*, including the body, head, pectoral fins, tail, and eyes. This image-based phenotyping approach offers clear advantages for fish morphological analysis. Relative to manual measurements and landmark-based geometric morphometrics, instance segmentation supports high-throughput processing of large image datasets under standardized acquisition conditions, reducing operator-dependent error and time requirements [52]. The method also shows high accuracy and reproducibility. YOLO-family models, including YOLOv8 and YOLOv11, have achieved high segmentation precision in fish-related applications, with mAP@50 values commonly exceeding 98%, while maintaining stable boundary detection across variable backgrounds, illumination conditions, and body postures [53]. Direct calculation of mask-derived area metrics enables the extraction of multidimensional descriptors linking regional proportions to overall body shape, providing standardized indices for the assessment of morphological plasticity, population differentiation, and environmental adaptation [21].

It should be noted that, although the present study operationally distinguishes individuals as sea-caught and farmed, the biological composition of sea-caught *L. crocea* in contemporary coastal fisheries is inherently complex. As acknowledged in the Introduction, sea-caught samples may include naturally reproducing individuals, stock-enhanced fish, and escapees from aquaculture cages. In this study, sea-caught individuals were classified based on capture location and fishing method rather than confirmed genetic origin. Consequently, some degree of phenotypic overlap between sea-caught and farmed groups cannot be excluded. This compositional uncertainty may partially reduce the apparent magnitude of morphological divergence, and thus the observed differences are likely conservative estimates of the true phenotypic contrast between purely wild and farmed populations. Future studies integrating genetic assignment, otolith microchemistry, or tagging-based approaches will be essential to refine population classification and strengthen biological interpretation.

Several limitations remain. Two-dimensional imaging approaches, including X-ray and visible-light photography, capture projected areas only and do not represent body thickness or three-dimensional volume, which may lead to underestimation of true morphological differences [54]. Area-based measurements depend on external calibration references, and variation in illumination or specimen posture can still introduce uncertainty. Model generalization also represents a challenge, as YOLO-based architectures require scenario-specific fine-tuning when applied across different imaging environments. Area-based phenotypes quantify structural variation effectively, but their relationships with swimming energetics, skeletal development, and behavioral performance remain largely correlational, limiting functional interpretation.

Although clear differences in external morphology were observed between sea-caught and farmed *L. crocea*, the interpretative scope of the area-based traits used in this study should be clearly defined. All morphological indices were derived from two-dimensional lateral projections and describe relative surface-area allocation among external body regions rather than direct functional performance. Consequently, associations with energy storage, locomotion, or swimming capacity represent morphological tendencies inferred from established ecological and biomechanical knowledge, not direct functional measurements. Two-dimensional projected area does not capture body thickness or three-dimensional mass distribution, which are critical for functional performance. Nevertheless, when applied consistently across large sample sizes, area-based phenotypes provide effective descriptors of structural divergence and long-term responses to contrasting rearing environments. Future studies integrating direct functional measurements with three-dimensional or volumetric imaging approaches will be essential for strengthening morphology–function interpretations.

Future studies may integrate multi-view reconstruction, three-dimensional photogrammetry, and CT or micro-CT imaging to complement volume-related traits. Combining deep-learning-based phenotyping with physiological and biomechanical experiments may further enable construction of a unified morphology–function–ecology framework [17]. Overall, the YOLOv11-Seg approach developed here enables high-throughput and reproducible quantification of external fish morphology and provides a practical technical pathway for intelligent phenotyping in ichthyological research.

### 4.3. Morphometric Data Analysis

This study showed that both total body area and the absolute areas of individual anatomical regions were larger in farmed *L. crocea*, whereas the relative proportions of locomotor structures, including the tail and pectoral fins, were reduced. This pattern indicates that aquaculture conditions favor energy assimilation and somatic growth while relaxing selective pressures associated with mobility. Farmed individuals therefore tend to increase trunk mass to support rapid growth, accompanied by reduced relative investment in propulsive structures that facilitate fast swimming, predator avoidance, and energy-efficient locomotion in natural environments.

This morphological pattern corresponds to well-documented differences between wild and farmed fish across marine species. Cultured fish commonly exhibit shorter and deeper body forms, characterized by increased body depth or girth, whereas wild individuals retain more streamlined shapes and relatively enhanced locomotor structures, such as larger caudal fins or narrower caudal peduncles. In Mediterranean gilthead sea bream (*Sparus aurata*) and European sea bass (*Dicentrarchus labrax*), body-shape metrics reliably distinguish wild from farmed individuals, and traits associated with swimming performance are consistently more pronounced in wild fish [55].

Within *L. crocea*, recent studies comparing geographically distinct wild populations have demonstrated that multivariate analyses based on external morphological indices, including body depth, body thickness, caudal peduncle ratios, and fin-area proportions, can effectively resolve population structure and inform germplasm conservation and selective breeding. The same analytical framework is applicable to evaluating divergence between farmed and wild groups. The area-based regional composition metrics quantified here align with the established trade-off between somatic growth and locomotor investment observed across marine fishes.

Together, these results provide quantitative evidence that morphological resource allocation is shaped by the combined effects of environmental conditions and artificial selection. Aquaculture environments characterized by abundant feed, restricted swimming space, and reduced predation pressure promote phenotypic investment in trunk enlargement, while locomotor structures become relatively diminished. This shift reflects functional reallocation toward growth-related traits under constrained activity regimes.

### 4.4. Applied Value of Instance-Segmentation Phenotypes

At the application level, the proposed “mask–metric–threshold” pipeline can be integrated into routine quality-control systems used by aquaculture enterprises. Composition indices, including Body/Total, Tail/Total, and Pectoral/Total, can function as online decision variables and early-warning indicators. These metrics can be linked to automated grading, harvest scheduling, and batch management, forming a unified workflow that combines segmentation with length and weight estimation and grading. Non-contact measurement and instance-segmentation-based size estimation have already been validated across multiple fish species, providing an engineering-ready basis for online quality control [56,57,58].

In breeding programs, phenotypic indicators derived from instance segmentation can be incorporated into broodstock screening and family-based evaluations, serving as a phenotype-support layer for multi-trait selection related to growth, disease resistance, and stress tolerance. This integration can enhance genetic gain and improve production stability. Recent reviews of *L. crocea* have summarized progress from germplasm conservation to marker-assisted and genomic selection, as well as intelligent monitoring approaches, outlining both methodological and industrial pathways for coordinated phenotype–genotype improvement [59,60].

In processing and market contexts, integrating area-based phenotypes into automated length and weight estimation, grading, and size-standardization workflows reduces subjective human error and increases production-line efficiency. When combined with predictive models for carcass traits and market attributes, these phenotypes can support a quantitative “image–specification–pricing” interface [56].

From a research perspective, linking area-based phenotypes with quality traits, including muscle physicochemical properties, texture, and nutritional composition, would enable integrated modeling frameworks for evaluating quality variation across aquaculture systems, such as deep-water and conventional net cages. This integrated evidence chain aligns with studies showing that environmental and hydrodynamic differences influence both body shape and muscle quality, supporting refined strategies for feeding management and density–flow optimization [61].

In ecological management and regulatory contexts, morphological profiling combined with multivariate discrimination supports escapee identification and origin tracing. When further integrated with genetic or geochemical markers, such as otolith elemental signatures, this approach provides a robust toolkit for stock assessment and risk mitigation, reducing risks of genetic introgression and ecological spillover [55].

Within the broader framework of intelligent aquaculture, instance-segmentation-derived phenotypes can function as a central linkage between smart monitoring, precision breeding, and quality management. These phenotypes interface with underwater vision systems for detection, feeding optimization, and health monitoring, while also connecting with breeding and processing metrics. This structure aligns with recent developments in computer-vision-based fish phenotyping and underwater segmentation, and is consistent with behavioral and ecological observations of *L. crocea* in hydrodynamic net-cage environments [62,63].

## 5. Conclusions

This study demonstrates that image-based instance segmentation can be effectively used to quantify external morphological traits in the large yellow croaker (*L. crocea*) in a standardized and reproducible manner. By combining visible-light imaging with automated segmentation and area-based measurements, we established a practical workflow for objective comparison of external body regions between sea-caught and farmed populations.

Clear morphological differences were identified between the two groups. Farmed individuals showed a greater proportional allocation to the trunk region, whereas sea-caught fish maintained relatively higher contributions from the tail and pectoral fins. These differences are consistent with contrasting ecological and rearing conditions and reflect long-term effects of domestication on morphological allocation. The use of area-based traits provides a quantitative description of such divergence without relying on labor-intensive manual measurements.

From an applied perspective, the proposed approach has potential value in aquaculture and fisheries management. Image-derived morphological indices may serve as auxiliary traits in breeding programs, support non-contact phenotypic monitoring during production, and assist in the identification of escapees in coastal fisheries. At the same time, the present study is limited to two-dimensional external morphology, and functional interpretations remain indirect.

Future research should therefore incorporate direct measurements of swimming performance, energetic expenditure, and biomechanical traits to strengthen links between morphology and function. Integration of multi-view imaging, three-dimensional reconstruction, and volumetric analyses would further improve the biological interpretation of image-based phenotypes. Together, these efforts will contribute to a more comprehensive understanding of domestication-driven morphological change and support the development of quantitative tools for intelligent aquaculture and fish phenomics.

## Figures and Tables

**Table 1:** Performance comparison of different object detection and instance segmentation models on the *L.* *crocea* dataset.

Model	Data Set	Precision (%)	Recall (%)	mAP@50 (%)	mAP@50–95 (%)
YOLOv11	test	98.53	99.24	98.97	83.24
YOLOv5	common COCO	96.60	98.00	97.90	78.50
Transformer-based DETR	test COCO	99.00	88.00	98.30	81.20
YOLOv8	yolov8	97.10	98.00	98.40	80.65

**Table 2:** Area and proportional composition of body regions in sea-caught and farmed *L. crocea*.

Group	Body (cm^2^)	Eyes (cm^2^)	Head (cm^2^)	Pectoral Fin (cm^2^)	Tail (cm^2^)	Total (cm^2^)
Sea-caught (Average)	20.64	0.31	5.22	1.91	2.40	30.47
Sea-caught (Proportion, %)	67.72	1.02	17.12	6.27	7.88	100
Farmed (Average)	14.65	0.19	3.15	0.99	1.22	20.20
Farmed (Proportion, %)	72.53	0.94	15.59	4.89	6.06	100

**Table 3:** Comparison of average surface area and proportional composition of different body parts between farmed and wild *L. crocea*.

Body Part	Farmed Average Area (cm^2^)	Farmed Proportion (%)	Wild Average Area (cm^2^)	Wild Proportion (%)	Difference Trend
Body	14.652	72.53	20.636	67.72	Wild ↑ (larger area, lower proportion)
Eyes	0.190	0.94	0.310	1.02	Wild ↑
Head	3.149	15.59	5.218	17.12	Wild ↑
Pectoral Fin	0.987	4.89	1.909	6.27	Wild ↑
Tail	1.223	6.06	2.400	7.88	Wild ↑

**Table 4:** Recognition performance of the YOLOv11 model on blind samples of *L. crocea*.

Identification Outcome	Number of Samples (*n*)	Percentage of Total Samples (%)
Correct identification	95	77.87
Unidentified	13	10.66
Misidentification	14	11.47

## Data Availability

The data supporting the findings of this study are available from the corresponding authors upon reasonable request. Due to institutional data management policies, the raw X-ray images and intermediate analysis files cannot be publicly shared but can be provided to qualified researchers upon request.
